# Could Anemia Impact the Severity of Infections? COVID-19 as an Example

**DOI:** 10.12688/f1000research.144790.1

**Published:** 2024-04-18

**Authors:** Sham ZainAlAbdin, Salahdein Aburuz, Amal Akour, Rami Beiram, Munther Alnajjar, Derar Abdel-Qader, Mosab Arafat, Anan Jarab, Mohammed Aburuz, Sara AlAshram, Sara AlJabi, Fatima AlSalama, Mohammed Al Hajjar

**Affiliations:** 1 Department of Pharmacology and Therapeutics, College of Medicine and Health Sciences, United Arab Emirates University, Al Ain, Abu Dhabi, United Arab Emirates; 2 Department of Clinical Pharmacy, The University of Jordan, Faculty of Pharmacy, Amman, Jordan; 3 Department of Clinical Pharmacy, American University of Madaba, Amman, Amman Governorate, Jordan; 4 Pharmacy Department, University of Petra, Amman, Amman Governorate, Jordan; 5 College of Pharmacy, Al Ain University, Al Ain, Abu Dhabi, United Arab Emirates; 6 Department of Clinical Pharmacy, Faculty of Pharmacy, Jordan University of Science and Technology., Irbid, 22110, Jordan; 7 The University of Jordan, Amman, Amman Governorate, Jordan; 8 Department of Pharmacy, Al Ain Hospital, Al Ain, Abu Dhabi, United Arab Emirates

**Keywords:** COVID-19, Anemia, Severity, ICU admission, Hospitalization, Mortality, Hemoglobin, Ferritin

## Abstract

**Background:**

The association between anemia and severity of infection as well as mortality rates among patients infected with COVID-19 has scarcely been studied. This is the first study UAE aimed to assess the influence of anemia on COVID-19 severity, ICU admission, and mortality rate.

**Methods:**

A retro-prospective chart review of hospitalized COVID-19 patients was conducted in a large COVID-19 referral hospital in UAE. The study included adult patients with confirmed COVID-19. Clinical and laboratory data, severity of the disease, ICU admissions, and mortality rates were analyzed and correlated to the presence of anemia among the patients.

**Results:**

A total of 3092 patients were included. 362 patients (11.7%) were anemic and most of the cases were between asymptomatic and mild COVID-19 (77.4%, n=2393). Among patients with anemia, 30.1% (n=109) had moderate to severe COVID-19. Statistically, anemia was associated significantly with a higher risk for severe COVID-19 outcome compared to nonanemic patients (AOR:1.59, 95% CI:1.24-2.04, p<0.001). Intensive care unit (ICU) admission was almost 3 times higher among anemic patients compared to nonanemic (AOR:2.83,95% CI:1.89-4.25, p<0.001). In addition, the overall mortality rate of 2.8% (n=87) was 2.5-fold higher in anemic than nonanemic patients (OR:2.56, CI: 1.49-4.06, p<0.001). Moreover, older age (≥48-year-old) and male gender were independent predictors for severe illness (Age: OR=1.26, CI:1.07-1.51, p=0.006; Gender: OR:1.43,CI:1.15-1.78, p<0.001)) and ICU admission (Age: OR:2.08, CI:1.47-2.94, p<0.001; Gender: OR: 1.83, CI:1.12-3.00, p=0.008) whereas only age ≥48 years old contributed to higher mortality rate (OR:1.60, CI:1.04-2.46, p=0.034).

**Conclusion:**

Anemia was a major risk factor for severe COVID-19, ICU admission and mortality among hospitalized COVID-19 patients. Thus, healthcare providers should be aware of monitoring the hematological parameters among hospitalized patients with COVID-19 and anemia to reduce the risk of disease complications and mortality. This association should also be considered in other infectious diseases.

## Introduction

Anemia is a global health concern affecting more than 1.62 billion people worldwide (approximately 24.8%). ^1^ The World Health Organization (WHO) defines anemia as a condition in which the number of red blood cells or the hemoglobin concentration within them is lower than normal. Low hemoglobin levels are considered at <120 g/L in females and <130 g/L in males. Based on hemoglobin level, severity of anemia can be categorized as mild (110-119 g/L for females and 110-129 g/L for males), moderate (80-109 g/L) or severe (less than 80 g/L). ^2^ Studies in the Arab region, assessing the clinical characteristics of COVID-19 patients, have not explored the prevalence of anemia nor tested its association with COVID-19 severity and mortality.

Coronavirus disease of 2019 (COVID-19) has become a global pandemic since its outbreak in Wuhan (Hubei Province, China) in December 2019. ^3^ According to the National Emergency Crisis and Disasters Management Authority (NCEMA), more than 700 thousand cases were confirmed, and 2135 deaths were reported in the United Arab Emirates (UAE) until November, 4 ^th^ 2021 and the rate of developing new cases has increased to 67.0% by 2023 although majority of the people are vaccinated against COVID-19. ^4^ Genetic mutations of severe acute respiratory syndrome coronavirus-2 (SARS-COV-2) resulted in the emergence of more contagious variants; hence, increasing cases on daily bases. ^5^ Severe infections, such as COVID-19, are associated with hyper-inflammatory state, which might cause alteration in iron homeostasis. ^6^^,^^7^ The main suggested mechanism was explained by increased iron acquisition and retention within macrophages and decreased intestinal absorption of iron. Subsequently, this reduces iron availability for erythropoiesis, decreasing the production of hemoglobin. Altogether, the inhibition of erythropoiesis by inflammatory markers, shortened half-life of erythrocytes and decreased biological activity of erythropoietin induce the development of anemia. In contrast to functional iron deficiency, which is associated with low ferritin levels and elevated transferrin levels, the anemia of inflammation is characterized by low levels of iron and transferrin or reduced saturation of transferrin with iron, while ferritin levels are normal or elevated. ^6^ Furthermore, the presence of anemia has been identified as a major risk factor for hospitalization and mortality in several chronic and inflammatory diseases. ^6^^–^^8^

Since systemic inflammation is a common manifestation among COVID-19 patients, the presence of anemia in COVID-19 patients may well enhance disease progression and severity. ^6^ However, the exact correlation is unclear, as whether it is a baseline characteristic of patients, or developed secondary to the inflammatory process of COVID-19. In this regard, studies have investigated the association between anemia and COVID-19 severity and mortality. However, there were some limitations in the literature regarding the prevalence and pathogenesis of anemia in COVID-19, as well as its impact on disease severity and mortality.

Anemia was one of the most common hematologic findings in severe or critically ill COVID-19 patients. ^9^ A study showed a severe disease presentation in terms of prolonged hospitalization and the need for ICU admission or mechanical ventilation. Generally, elevated ferritin levels were related to disease severity. However, anemia was not associated with higher mortality rate. ^6^ However, a recent study found that elevated ferritin levels was a predictor for mortality in COVID-19 patients. ^10^

Recent data showed that anemia was significantly associated with severe illness and higher mortality rate, ^8^^,^^11^ especially if anemia was present upon admission. ^6^^,^^8^ Moreover, Tremblay *et al.* found that elevation of red blood cell distribution width (RDW) was a poor prognostic factor in hospitalized patients. ^12^ RDW has been validated as a strong predictor of 30-day mortality, reflecting overall inflammation and oxidative stress. ^13^ Another study reported that anemia induced the development of severe COVID-19 pneumonia. ^14^ All these studies concluded that anemia could be a predictive factor of increasing COVID-19 severity and mortality. However, the available data regarding the association between anemia and COVID-19 severity and mortality are scarce and controversial. Moreover, studies evaluating parameters of severe outcomes failed to demonstrate truly this association in a large considerable sample size. ^15^^–^^17^

Remarkably, all the reviewed studies have not clearly classified cases in terms of severity (mild/moderate/severe) or evaluated the actual association between anemia and severity, ICU admission and mortality. Therefore, further studies are required to confirm this association in a larger cohort with definite stratification of severity per se. This is the first study aiming to assess the influence of anemia on COVID-19 severity, ICU admission and mortality rate in hospitalized patients in the UAE.

## Methods

### Study design, participants, and setting

This is a retrospective observational study carried out on adult patients (≥18 years old) with a confirmed diagnosis of COVID-19, as measured using polymerase chain reaction (PCR) testing on a nasopharyngeal swab, who were admitted to a large government tertiary care center in Al-Ain city, UAE between March and June 2020. All adult patients diagnosed with COVID-19 during the study period were eligible for inclusion.

The following were the exclusion criteria

1. Pediatrics (Age <18 years old)
2. Pregnant patients
3. Patients admitted for Surgery
4. Patients with Co-infection with other diseases
5. Patients with Cancer

### Ethical consideration

This study was approved by Covid-19 IRB committee at the Department of Health in the United Arab Emirates (reference number: DOH/CVDC/2020/1121, 3 ^rd^ of June 2020). Patients’ information was kept confidential and authors didn’t have access to information that could identify individual participants during or after data collection. Study was performed in accordance with relevant guidelines and regulations. This was a retrospective review patients’ files and therefore consent form is waived and not required.

### Data collection

During the period from June to July 2020, an IT engineer retrieved all the patients’ data from the hospital electronic databases for patients admitted from March to June 2020 in an Excel sheet. During the period from July to September 2020, two of the research investigators revised, cleaned, and coded the data independently to check for inter-rater reliability. Any disagreement was discussed between the two investigators. The data was accessed for the purpose of this research during the period from January-June 2022. The collected data encompassed demographic information (age, gender, and BMI) in addition to the death rate (calculated by dividing the number of dead cases by total number of the study sample) and presence of anemia for the purpose of our study. Baseline vital signs upon admission (heart rate, respiratory rate, oxygen saturation, systolic, and diastolic blood pressure) in addition to the most relevant laboratory parameters to anemia, such as (hemoglobin, hematocrit, ferritin, D-dimer, and alkaline phosphatase) were reviewed and analyzed.

Additionally, clinical manifestations on admission (cough, shortness of breath, fever, sore throat, pain, nausea, vomiting and diarrhea), disease severity (asymptomatic, mild, moderate, severe) related to COVID-19 infection were also presented in this study. The disease severity of COVID-19 was categorized according to the guidelines for diagnosis and treatment of COVID-19 published by the National Institute of Health (NIH) ( *Overview of COVID-19*). ^18^ The NIH has categorized the severity of COVID-19 from no symptoms to critically ill cases into 5 categories: asymptomatic, mild, moderate, severe, and critically illness. However, the authors of this study classified the severity of COVID-19 into two categories since the distribution of patients in these categories is inconsistent and for better tabulation of the findings as follows: 1) Asymptomatic/mild illness: those with confirmed COVID-19 with or without the typical symptoms of the disease with no evidence of dyspnea or abnormal chest imaging, and 2) Moderate to severe/critical illness: those with typical symptoms, evidence of dyspnea, PaO _2_ of ≥94%, and abnormal chest imaging, with or without respiratory failure/multiple organ dysfunction.

### Definitions

Anemia was defined as hemoglobin (Hgb) levels <12.0 g/dL in females and <13.0 g/dL in males as per the study site reference values. Anemia was further categorized based on hemoglobin levels as follows: mild: 10.0 g/dL to lower limit of normal range (12.0 g/dL in females and 13.0 g/dL in males), moderate: 8.0 to 10.0 g/dL, and severe: below 8 g/dL. ^14^ Anemia diagnosis was also confirmed from the hospital database.

### Study outcomes

The primary outcome of this study was to evaluate the association between anemia and COVID-19 severity, ICU admission and mortality in hospitalized COVID-19 patients. A secondary outcome was to investigate the effect of other demographic factors including age and gender on COVID-19 outcomes (disease severity, ICU admission rate, and mortality rate).

### Statistical analysis

The Statistical Package for the Social Sciences (IBM- SPSS, version 26.0) was used for the statistical analysis of data. Mean and standard deviation (SD) were calculated to present parametric data, while median and interquartile range were calculated for non-parametric data. Categorical variables were described as frequencies and percentages.

Chi-square test or Fisher’s exact test was calculated to assess the association between anemia and severity of symptoms (stratified as asymptomatic/mild and moderate/severe cases for patients with or without anemia). Similarly, the ICU admissions and mortality rate were assessed and compared for and between patients with and without anemia using the abovementioned statistics. The differences in laboratory parameters between patients with or without anemia were analyzed using the independent t-test or Mann-Whitney test. A p-value less than 0.05 indicated a statistically significant difference with a confidence interval of 95.0%. Additionally, multivariable logistic regression analysis was performed to confirm the influence of anemia on disease severity, ICU admission and mortality.

## Results

Table 1 represents the differences in demographics and clinical characteristics of the patients with and without anemia. The study included a total of 3092 patients. The average age of all patients (including anemic and non-anemic) was 44.29±13.42 years old and majority of them were males (77.8%, n=2407). In addition, 11.7% of total patients were previously diagnosed with anemia (n=362).

**Table 1.** Demographic and clinical characteristics of the patients (n=3092).

Variables	Non-anemic (n=2730)	Anemic (n=362)	p-value
**Gender, n (%)**			
Male (n=2407)	2188 (90.9)	219 (9.1)	**<0.001**
Female (n=685)	542 (79.1)	143 (20.9)	
**Age, n (%)**			
18-47 yo (n=1902)	1733 (91.1)	169 (8.9)	**<0.001**
≥48 yo (n=1190)	997 (83.8)	193 (16.2)	
**BMI, n (%)**			
<30 kg/m ^2^ (n=2442)	2182 (89.4)	260 (10.6)	**<0.001**
≥30 kg/m ^2^ (n=650)	548 (84.3)	102 (15.7)	
**Vital Signs (Mean±SD)**			
Age (years)	43.6±12.9	49.7±15.9	**<0.001**
Heart rate (beats/min)	91.25±14.66	92.95±14.53	**0.038**
Respiratory rate (breaths/min)	19.45±4.74	20.72±5.74	**<0.001**
Systolic Blood pressure (mmHg)	136.18±17.89	132.60±20.69	**0.002**
Diastolic Blood pressure (mmHg)	82.15±11.61	77.09±13.86	**<0.001**
**Lab parameters Mean (SD) or Median (IQ range)**			
D-dimer (μg/mL)	0.82±2.44	1.40±2.98	**0.001**
HCT (%)	M: 0.42 (0.04)	M: 0.38 (0.06)	**<0.001**
	F: 0.37 (0.04)	F: 0.34 (0.05)	
Hgb (g/dL)	M: 14.49 (1.46)	M: 12.60 (2.25)	**<0.001**
	F: 12.48 (1.32)	F: 10.97 (1.86)	
Oxygen saturation (%)	97.84±3.66	96.30±6.39	**<0.001**
BMI (kg/m ^2^)	27.51±7.56	28.51±6.97	**0.011**
HBA1c	8.60±2.30	8.02±2.09	**0.044**
Ferritin (mg/L)	M: 416.00 (244.00-745.00)	M: 630.00 (331.00 – 1288.00)	**<0.001**
	F: 123.00 (54.00-249.00)	F: 170.00 (54.00-503.00)	
INR	1.02±0.55	1.16±0.87	0.374

Univariate analysis of the biodata showed that anemia was higher among females compared to males (20.9% and 9.1%; respectively, p<0.001). In addition, patients aged 48 years old and older were more likely to present with anemia compared to patients aged between 18 and 47 years old (16.2% and 8.9%; respectively, p<0.001). Furthermore, anemia was more prevalent among patients with BMI ≥30 kg/m ^2^ when compared to patients with lower BMI (15.7% and 10.6%; respectively, p<0.001).

Upon evaluating the relationship between anemia and certain hematological parameters, it was found that D-dimer and ferritin were significantly higher in patients with anemia compared to the non-anemics (p<0.001). In contrast, hemoglobin and hematocrit levels were remarkably lower in patients with anemia (p<0.001) compared to the non-anemics. Further details of the differences of vital signs and laboratory parameters among patients with and without anemia are illustrated in Table 1.

**Figure 1.**
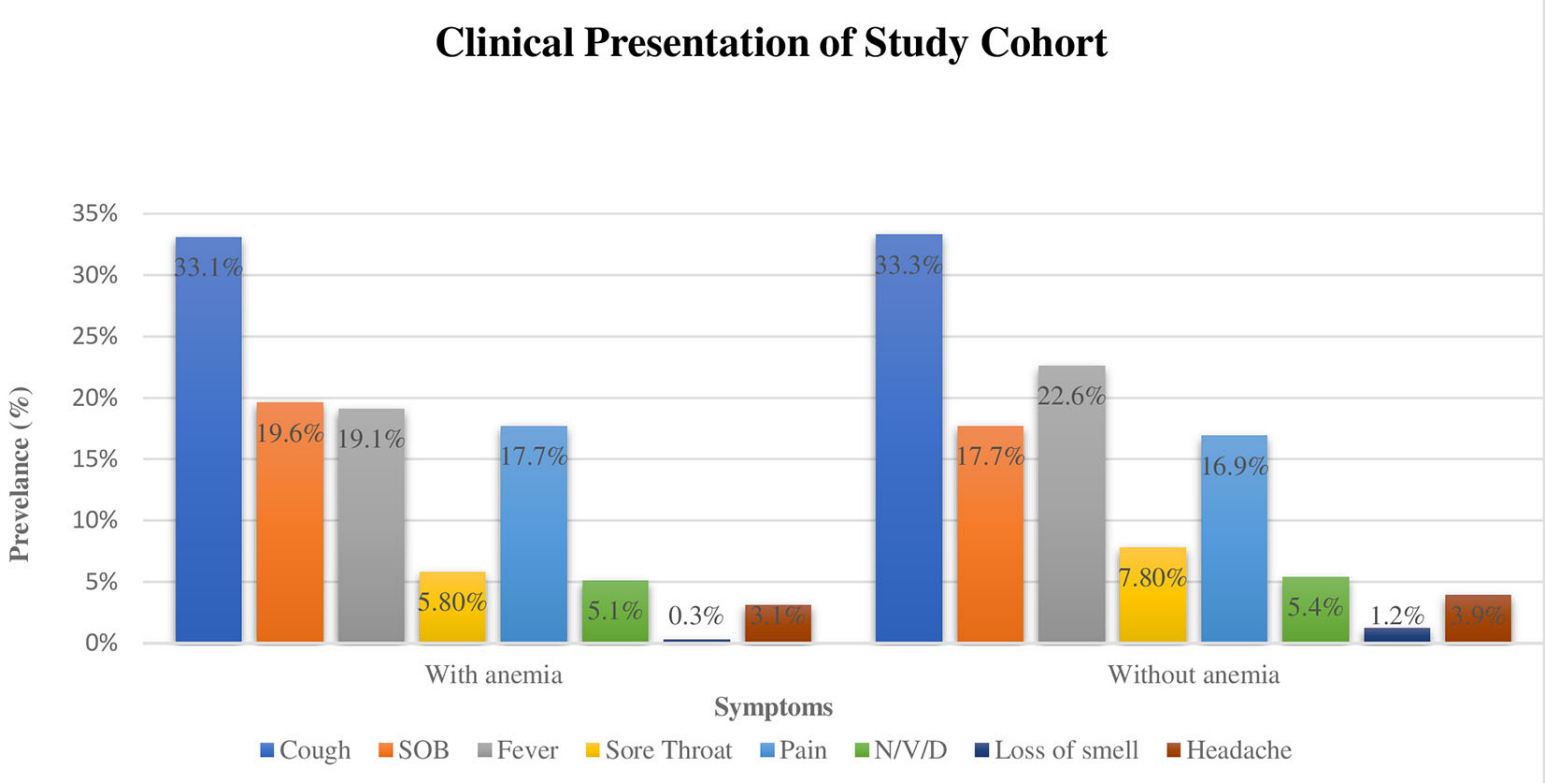
Clinical Presentation among anemic and non-anemic COVID-19 patients.

The majority of patients (77.4%, n=2393) were asymptomatic or had a mild form of the infection. Figure 1 shows the clinical presentation of patients with COVID-19. Cough was the most reported symptom by the patients followed by fever, and dyspnea. Other symptoms, such as loss of smell and taste were rarely reported. Regarding the clinical presentation of symptoms among anemic and nonanemic patients, no statistical difference was found.

Table 2 represents the Bivariate analysis of patients’ characteristics and COVID-19 outcomes (severity of COVID-19, ICU admissions, and mortality rate). The risk of developing moderate to severe symptoms was significantly higher when patients presented with anemia, male gender, and age ≥48 years old. In addition, ICU admission among anemic patients was almost 3 times higher than non-anemic patients, was also more than 2 times higher in patients aged ≥48 years old compared to young-age patients, and 1.5 times higher in males compared to females. Moreover, anemia was significantly associated with over 2.5 times risk of death, as 6.1% of anemic cases died compared to 2.4% of nonanemic cases. Also, patients aged ≥48 years old had almost 2 folds higher risk for mortality compared to younger ages. Further details are illustrated in Table 2.

**Table 2.** Bivariate analysis of patients’ characteristics and COVID-19 outcomes.

COVID-19 Outcomes	Independent variables	% (n/N)	Adjusted (OR)	Confidence Interval (CI)	P- value
**Severity (Moderate to Severe)** 22.6% (669/3092)	***Presence of Anemia***				
	Anemic	30.1% (109/362)			
	Non-anemic	21.6% (590/2730)	1.56	1.23-1.99	**<0.001**
	***Gender***				
	Female	19.0% (130/685)			
	Male	23.6% (569/2407)	1.32	1.07-1.64	**0.005**
	***Age***				
	18-47 YO	20.7% (394/1902)	1.32	1.11-157	**<0.001**
	≥ 48 YO	25.6% (315/1190)			
**ICU Admission** 4.6% (142/3092)	***Presence of Anemia***				
	Anemic	10.2% (37/362)			
	Non-anemic	3.8% (105/2730)	2.85	1.92-4.21	**<0.001**
	***Gender***				
	Male	5.0% (120/2407)	1.58	1.00-2.51	**0.028**
	Female	3.2% (22/685)			
	**Age**				
	18-47 YO	3.2% (60/1902)	2.27	1.62-3.20	**<0.001**
	≥ 48 YO	6.9% (82/1190)			
**Mortality** 2.8% (87/3092)	***Presence of Anemia***				
	Anemic	6.1% (22/362)			
	Non-anemic	2.4% (65/2730)	2.65	1.61-4.36	**<0.001**
	***Age***				
	18-47 YO	2.2% (42/1902)	1.74	1.14-2.67	**0.007**
	≥ 48 YO	3.8% (45/1190)			

Upon classifying patients anemia into normal, mild and moderate to severe; severity of COVID-19 was higher in anemic compared to normal patients (p <0.001). On the other hand, moderate-to-severe anemia was associated with greater ICU admissions and mortality rates (p <0.001) as presented in Table 3.

**Table 3.** Association between anemia severity and COVID-19 outcomes (n=3092).

Covid 19 outcomes	Severity of anemia	% (n/N)	P-Value
***Moderate to Severe Disease***	Normal*	21.6% (590/2730)	**<0.001**
	Mild	30.3 (90/297)	
	Moderate to Severe	29.3 (19/65)	
***ICU Admission***	Normal*	3.8% (105/2730)	**<0.001**
	Mild	9.1% (27/297)	
	Moderate to Severe	15.4% (10/65)	
***Mortality***	Normal*	2.4% (65/2730)	**<0.001**
	Mild	5.4% (16/297)	
	Moderate to Severe	9.2% (6/65)	

Multivariable logistic regression analysis of anemia and health outcomes among hospitalized patients with COVID-19 is shown in Table 4. After adjustment of gender, ages, BMI and anemia, anemia was shown to be a significantly independent risk factor for severe symptoms (AOR: 1.59, 95% CI: 1.24-2.04, p <0.001), ICU admission (AOR: 2.83, 95% CI: 1.89-4.25, p <0.001), and higher mortality rate (AOR: 2.46, 95% CI: 1.49-4.06, p <0.001). In addition, male gender and age (≥48-year-old) were independent risk factors that significantly contributed to developing moderate severe COVID-19 and increasing the rate of ICU admissions. Also, age (≥48 years old) was an independent risk factor for mortality as well.

**Table 4.** The logistic regression analysis of anemia and health outcomes among hospitalized patients with COVID-19.

Variables	Adjusted Odds Ratio (AOR)*	Confidence Interval (CI)	P-value
**Severity**			
Anemia	1.59	1.24-2.04	<0.001
Age (≥48-year-old)	1.27	1.07-1.51	0.006
Gender (male)	1.43	1.15-178	<0.001
**ICU admission**			
Anemia	2.83	1.89-4.25	<0.001
Age (≥48 yo)	2.08	1.47-2.94	<0.001
Gender (male)	1.83	1.12-3.00	0.008
**Mortality**			
Anemia	2.46	1.49-4.06	<0.001
Age (≥48-year-old)	1.60	1.04-2.46	0.034

## Discussion

To the best of our knowledge, our study is the first comprehensive, decently sized study addressing the potential association between anemia and clinical outcomes in hospitalized COVID-19 patients in the United Arab Emirates. These outcomes included disease severity, risk of ICU admission, and mortality. Additionally, the study explored possible correlation between certain demographic variables and the disease outcomes. Based on the data collected from 3092 hospitalized adult patients in one of the largest hospitals in UAE, majority of the patients were males, 11.7% of whom were anemic. Compared to findings of previous studies, our findings indicated lower prevalence of anemia in the studied sample (11.4%), whereas other studies reported a higher prevalence of anemia ranging from 24.7%-35.5% among hospitalized COVID-19 patients. ^6^^,^^11^ This could be explained by the predominance of males and young age (<48 years old) patients, who are considered to be at lower risk of suffering from anemia.

The prevalence of anemia was higher in females. Generally, anemia is reported to be more prevalent in females and lower hemoglobin values are seen among females compared to males even in cases without anemia, which is attributed to low iron intake and reproductive issues such as menstruation, pregnancy, and lactation. ^19^ Interestingly, studies evaluating anemia in COVID-19 patients found a higher prevalence of anemia within the male population. ^20^^,^^21^ On the other hand, other studies found a higher prevalence of anemia within females. ^11^^,^^14^ Of note, however, these studies failed to demonstrate a significant association between anemia and gender. This variation from the well documented fact about anemia prevalence could be attributed to the lack of distinction between the commonly encountered iron deficiency anemia and anemia of inflammation typically linked to COVID-19.

Our modelling analysis showed that patients aged ≥ 48 years old had higher prevalence of anemia. This is in agreement with many previous studies that demonstrated a higher prevalence of anemia with advancing age. ^11^^,^^14^^,^^20^^,^^21^ Interestingly, while Chen et al. demonstrated an increasing anemia prevalence with age, the prevalence then decreased at age of 80 and above years old; however, a small number of cohort was reported in that study. ^14^ The relationship between anemia and aging could be explained by common nutritional deficiencies (iron, folate, or vitamin B12), typically encountered with elderly or anemia of chronic diseases, and sometimes due to unknown reason. This is mainly because with aging there seems to be a progressive resistance of bone marrow erythroid progenitors to erythropoietin, and a chronic subclinical pro-inflammatory state, resulting in anemia. ^19^

Our study showed that most of COVID-19 cases with anemia were associated with high serum ferritin, which is mainly attributed to the fact that ferritin levels elevate dramatically during infection or inflammation. ^22^ This is because ferritin by itself is a key biomarker in inflammatory and pathological conditions, mainly because it leaks from damaged cells. ^23^ Basically, males have higher ferritin levels than females throughout their adulthood in which they peak between 30 to 39 years and stay constant until their 70’s. Whereas, females have relatively low ferritin levels, beginning to rise after menopause. ^22^ Few studies highlighted the correlation between serum ferritin levels and COVID-19 clinical characteristics and disease outcomes. Cheng *et al* found that ferritin levels generally varied based on COVID-19 severity and presence of comorbidities. Ferritin levels were significantly elevated in patients with COVID-19 and other comorbidities -including anemia- rather than COVID-19 alone. This could be probably due to the presence of concurrent inflammatory and pathological reactions. ^24^ Similarly, this finding was also illustrated in a previous observational study which reported that anemia was highly prevalent among 206 hospitalized COVID-19 patients. ^20^

Although COVID-19 is a primary respiratory illness, it also affects multiple organs and results in systemic complications, such as thrombotic disorders and coagulopathies. Several studies reported significant elevation in D-dimer among hospitalized COVID-19 patients, reflecting the remarkable prevalence of thrombotic disorders in this population. Thus, elevation of D-dimer could be a potential biomarker of COVID-19 poor prognosis. ^25^ A cut-off value of D-dimer has not yet been established to predict mortality in patients with COVID-19, but one study published lately in 2021 described a cut-off point of 1.5μg/ml (sensitivity 70.6%, specificity 78.4%). ^25^ Yet only one study compared the levels of D-dimer in anemic versus non-anemic COVID-19 patients. They found that D-dimer was higher in patients with moderate/severe anemia compared to mild anemia. ^11^ This is in line with our findings that D-dimer levels were higher in the anemic patients’ group (p<0.001). There is a lack of clear justification behind the additional elevation of D-dimer among COVID-19 anemic cases, and further studies are required to understand this finding manifestly.

The present study reported that majority of COVID-19 patients were asymptomatic or had mild symptoms of COVID-19. However, patients with moderate or severe symptoms presented mainly with cough and fever. Likewise, a systematic review of 152 studies indicated that fever and cough were highly prevalent among COVID-19 patients. ^26^ However, some reports revealed that in addition to cough and fever, a sudden loss of smell or taste is a common manifestation of the disease. ^27^^,^^28^

An interesting finding of this study was the significant association between anemia and the risk of developing moderate to severe symptoms *.* This finding is in agreement with previously published studies that found a significant effect of anemia on disease severity related to the severe inflammatory response. ^11^^,^^29^ Additionally, several studies that explored the parameters of developing severe disease outcomes failed to demonstrate this association with a considerable sample size. ^15^^–^^17^ Fan (2020) investigated 69 patients and found that lymphopenia and elevated LDH were associated with higher rates of ICU admissions. ^15^ In addition, Henry *et al* pooled data into a meta-analysis from 21 studies with small sample sizes; 18 studies of which inspected the severity, and the other 3 assessed the mortality rate of COVID-19 by studying several hematological parameters. Although this analysis reported a significant decline in hemoglobin levels among patients with a severe form of the disease, it did not assess anemia as a determinant factor of COVID-19 severity. ^16^

Besides, findings of this study showed almost 3-fold increase in the risk of ICU admission in COVID-19 patients with anemia compared to their counterparts. This was further proven by the results of a multivariable logistic regression after adjustment for covariates. Our results concurred with a prospective study conducted in the Middle East with a decently sized cohort (n=1274), which supports our finding that anemia is an independent risk factor for ICU admission among patients with COVID-19 (p<0.001). ^21^ On the other hand, Cai *et al.* investigated factors associated with ICU admission and failed to find an association between hemoglobin levels and risk of being admitted to the ICU. ^30^ Such results could be attributed to the small sample size of the study (n=96). In the same manner, Bellman-Weiler *et al.* found no significant relationship between anemia and ICU admission, but concluded that alterations in iron homeostasis -as a higher ferritin/transferrin ratio- reflected further risk of advanced disease and predicted the need for ICU admission. ^6^ However, these findings were limited to those without iron metabolism variability, which resulted in a selection bias and a smaller sample included in the multivariate analysis. Moreover, according to Bellman-Weiler *et al.* hemoglobin -as a variable- was not a significant predictor in their multivariate regression analysis, illustrating a probable residual confounding for anemia classification. Furthermore, similar studies recognized the relationship between the presence of anemia and high mortality rate amongst ICU-admitted COVID-19 patients. However, these studies either did not assess ICU admission as a final outcome due to the rapid turnover (discharge or death) ^8^^,^^20^ nor tested if the presence of anemia had an effect on admission to the ICU. ^29^^,^^31^

According to findings of the current study, anemia was associated with a high mortality rate among hospitalized COVID-19 patients compared to non-anemic cases with COVID-19. This observation was further reaffirmed using a multivariable logistic regression analysis, after adjusting for covariables that could contribute to this finding. The results of this study showed that anemia was an independent predictive risk factor for death in COVID-19 patients. Similarly, most studies investigated the relationship between anemia and mortality in COVID-19 patients, and all of them came to the agreement that anemia significantly contributes to a higher mortality rate. Correspondingly, a single center retrospective study assessed the impact of anemia on mortality rate among 333 hospitalized COVID-19 patients with anemia. The study has found that more than half of these patients were significantly at high risk of all-cause mortality. Moreover, AbuRuz, S *et al*, Bellman *et al*., Algassim et al, Dinevari *et al*, Tremblay *et al*, and Oh *et al*. showed similar results and further confirmed this association. ^6^^,^^12^^,^^21^^,^^31^^,^^32^ In addition, Bellman *et al* further analyzed this association and compared the mortality rate between mild and moderate/severe anemia. Accordingly, only moderate/severe anemia was significantly associated with a higher mortality rate. Furthermore, their study examined this association after stratifying the subjects by anemia type, as anemia of functional iron deficiency vs. anemia of inflammation. They concluded that anemia of iron deficiency was not associated with higher mortality rates compared to non-anemic cases. ^6^ On the other hand, Tremblay *et al.* reported that even mild anemia is a predictive risk factor of COVID 19 mortality. ^12^

Considering other factors, multivariable regression analysis revealed that male gender, older age (≥48-year-old), significantly increased the severity of COVID-19 and ICU admissions. However, only older age was found to be an independent risk factor in addition to anemia for mortality. Previous studies evaluating the clinical impact of anemia did not evaluate the impact of demographics on the disease outcomes, rather they outlined the relationship between demographic factors and anemia. ^19^ Available studies simply reported the prevalence of anemia in each demographic group, and tested if a significant difference was observed. ^21^

### Strength & limitations

Our study is the first study in the Gulf region that spotted the light on the association between anemia and COVID-19 outcomes in terms of severity, ICU admission and mortality. We included a large sample size of COVID-19 patients in a hospital that was specialized for COVID-19 cases. We studied the association between anemia and COVID-19 severity, ICU admission and mortality by conducting a multivariable regression analysis with adjustment of certain covariables that could affect the association. Another point that highlighted the novelty of our study was that we considered other demographic factors and we tested their impact on disease severity, mortality and ICU admission using a multivariable model analysis. In addition, we measured several hematological parameters including hemoglobin, hematocrit, D-dimer and ferritin which reflected the level and significance of anemia in COVID-19 patients. Finally, our population included patients at younger ages compared to previously presented studies, making the influence of age and other comorbidities on the results less significant, since not all of the patients were elderly.

The present study had several limitations. First, this was a retrospective observational study; therefore, we were unable to accurately control exposure factors, covariates, and potential confounders. Second, it was a single center study, hence, it would be difficult for the results to be emphasized and generalized amongst other areas in the region. Third, our data were not longitudinal; we had no information about hemoglobin levels before infection and we were unable to track the changes in hematological parameters during hospitalization. Fourth, we did not collect and analyze the clinical interventions and therapeutic information related to anemia in COVID-19 patients. These data could have provided a valuable justification for the normalization of hemoglobin and hematocrit levels among patients. Lastly, we did not classify and clearly define anemia based on severity, and the etiology of anemia was not distinguished, whether it is a functional iron deficiency or anemia of inflammation. This limited our interpretations and affected the causal association between anemia and COVID-19 outcomes.

## Conclusion

Anemia was a common clinical manifestation among COVID-19 patients. This study highlighted the association between anemia and COVID-19 clinical outcomes. Analysis of several hematological parameters illustrated that anemia was an independent predictive factor for poor COVID-19 outcomes. Other demographic factors, such as age and gender had an impact on the clinical outcomes in COVID-19 patients. These results could be a guidance for clinicians in triaging hospitalized COVID-19 patients upon admission by considering their clinical parameters, particularly, hemoglobin levels. In turn, this could enhance the healthcare team’ decision for early, intensive therapeutic interventions for certain patient and tailor a patient-specific treatment plan. Further studies with more comprehensive follow-up of COVID-19 patients with anemia, stringent designs and encompassing multiple centers are needed to confirm causality and support generalizability and possibly develop a guideline for management of anemia in COVID-19 patients. This association should also be considered in other infectious diseases.

## Author contribution

Sham ZainAlAbdin: Conceptualization, Formal Analysis, Investigation, Methodology, Writing – original draft, Writing – review & editing

Salahdein AbuRuz: Conceptualization, Formal Analysis, Investigation, Methodology, Writing – original draft, Writing – review & editing, Supervision

Amal Akour: Conceptualization, Formal Analysis, Methodology, Writing – original draft, Writing – review & editing,

Rami Beiram: Conceptualization, Methodology, Writing – original draft, Writing – review & editing

Munther Alnajjar: Conceptualization, Methodology, Writing – original draft, Writing – review & editing

Derar Abdel-Qader: Conceptualization, Methodology, Writing – original draft, Writing – review & editing

Mosab Arafat: Conceptualization, Methodology, Writing – original draft, Writing – review & editing

anan jarab: Conceptualization, Methodology, Writing – original draft, Writing – review & editing

Mohammed Aburuz: Data Curation, Writing – Original Draft Preparation, Writing – Review & Editing

Sara AlAshram: Conceptualization, Methodology, Writing – original draft, Writing – review & editing, Data curation

Sara AlJabi: Conceptualization, Methodology, Writing – original draft, Writing – review & editing, Data curation

Fatima AlSalama: Conceptualization, Methodology, Writing – original draft, Writing – review & editing, Data curation

Mohammed Al Hajjar: Conceptualization, Methodology, Writing – original draft, Writing – review & editing, Data curation

## Data Availability

Figshare: Could anemia impact the severity of infections? COVID-19 as an example. https://doi.org/10.6084/m9.figshare.24511999.v1 The project contains the following underlying data:
• Covid anemia.xlsx Data are available under the terms of the Creative Commons Attribution 4.0 International license (CC-BY 4.0). Repository: STROBE checklist for ‘Could anemia impact the severity of infections? COVID-19 as an example”. https://doi.org/10.5281/zenodo.10907120 Data are available under the terms of the Creative Commons Zero “No rights reserved” data waiver (CC0 1.0 Public domain dedication).
