# Supplementary material for: Could Anemia Impact the Severity of Infections? COVID-19 as an Example
Source: F1000Res. 2024 Nov 5;13:295. Originally published 2024 Apr 18. [Version 2] doi: 10.12688/f1000research.144790.2 (PMC11615857; doi:10.12688/f1000research.144790.2)
Supplement: Peer review history

# Reviewer report: Reviewer response for version 2

**DOI:** 10.5256/f1000research.174189.r337890

**Published:** 2024-11-27

**Authors:** Joan-Lluis Vives-Corrons

**Affiliations:** 1 Red Cell Pathology and Haematopoietic Disorders (Rare Anaemias Unit), Institute for Leukaemia Research Josep Carreras (IJC), Barcelona, Spain

In my opinion, the authors have effectively addressed all the comments and suggestions provided, and accordingly, successfully incorporated them into the updated manuscript.

Is the work clearly and accurately presented and does it cite the current literature?

Yes

If applicable, is the statistical analysis and its interpretation appropriate?

I cannot comment. A qualified statistician is required.

Are all the source data underlying the results available to ensure full reproducibility?

Yes

Is the study design appropriate and is the work technically sound?

Yes

Are the conclusions drawn adequately supported by the results?

Yes

Are sufficient details of methods and analysis provided to allow replication by others?

Yes

Reviewer Expertise:

NA

I confirm that I have read this submission and believe that I have an appropriate level of expertise to confirm that it is of an acceptable scientific standard.

# Reviewer report: Reviewer response for version 2

**DOI:** 10.5256/f1000research.174189.r337891

**Published:** 2024-11-14

**Authors:** John McDonnell

**Affiliations:** 1 Pediatric Allergy and Immunology, Cleveland Clinic Children's Hospital, Cleveland, OH, USA

These changes look good to me.

Is the work clearly and accurately presented and does it cite the current literature?

Yes

If applicable, is the statistical analysis and its interpretation appropriate?

Partly

Are all the source data underlying the results available to ensure full reproducibility?

Yes

Is the study design appropriate and is the work technically sound?

Yes

Are the conclusions drawn adequately supported by the results?

Partly

Are sufficient details of methods and analysis provided to allow replication by others?

Partly

Reviewer Expertise:

immunology, statistical methods

I confirm that I have read this submission and believe that I have an appropriate level of expertise to confirm that it is of an acceptable scientific standard.

# Reviewer report: Reviewer response for version 1

**DOI:** 10.5256/f1000research.158636.r280551

**Published:** 2024-07-09

**Authors:** John McDonnell

**Affiliations:** 1 Pediatric Allergy and Immunology, Cleveland Clinic Children's Hospital, Cleveland, OH, USA

This is an interesting study about the role of anemia in COVID-19 infection. It is overall well-written and scientifically important, but there are many small wording issues that if corrected will greatly enhance its clarity and readability.

 Additionally, there are a few more substantial statistical issues to consider. Because these affect the science behind the paper, I believe that they are very important for the authors to consider and address, even more so than the minor quibbles I list below about wording and grammar. Therefore, I have put these in bold.

 The most substantial statistical issue I have relates to the results section, where table 3 is referenced.

**Author List**:

 - The name "anan jarab" is not capitalized.

 - *Recommendation*: Ensure consistent capitalization in the author list, e.g., "Anan Jarab”, unless there is a specific reason not to.

**Abstract**:

**Background**:

 - The sentence "This is the first study UAE aimed to assess the influence of anemia on COVID-19 severity, ICU admission, and mortality rate." is unclear.

 - *Recommendation*: Revise to "This is the first study from the UAE aimed to assess the influence of anemia on COVID-19 severity, ICU admission, and mortality rate."

**Methods**:

 - The term "retro-prospective chart review" is confusing.

 - *Recommendation*: Use "retrospective chart review" for clarity.

**Introduction**:

 - this is a run-on sentence and is hard to understand -  "According to the National Emergency Crisis and Disasters Management Authority (NCEMA), more than 700 thousand cases were confirmed, and 2135 deaths were reported in the United Arab Emirates (UAE) until November, 4th 2021 and the rate of developing new cases has increased to 67.0% by 2023 although majority of the people are vaccinated against COVID-19."

 - *Recommendation*: Break this into two sentences for clarity: "According to the National Emergency Crisis and Disasters Management Authority (NCEMA), more than 700 thousand cases were confirmed, and 2135 deaths were reported in the United Arab Emirates (UAE) until November 4th, 2021. The rate of new cases increased by 67.0% by 2023, despite the majority of the population being vaccinated against COVID-19."

 -This seems to be the wrong “basis”: "Genetic mutations of severe acute respiratory syndrome coronavirus-2 (SARS-COV-2) resulted in the emergence of more contagious variants; hence, increasing cases on daily bases."

 - *Recommendation*: Correct the word "bases" to "basis": "Genetic mutations of severe acute respiratory syndrome coronavirus-2 (SARS-COV-2) resulted in the emergence of more contagious variants, hence increasing cases on a daily basis."

 - There is redundant wording in this sentence: "Moreover, studies evaluating parameters of severe outcomes failed to demonstrate truly this association in a large considerable sample size."

 - *Recommendation*: Simplify to avoid redundancy: "Moreover, studies evaluating parameters of severe outcomes failed to demonstrate this association in a large sample size."

**Ethical Considerations**:

 - The wording here is problematic, as past and present tenses are mixed. The use of contractions is probably best avoided in a scientific paper: "Patients’ information was kept confidential and authors didn’t have access to information that could identify individual participants during or after data collection. Study was performed in accordance with relevant guidelines and regulations. This was a retrospective review patients’ files and therefore consent form is waived and not required."

 - *Recommendation*: Revise for consistent tense and avoid contractions: "Patients’ information was kept confidential, and the authors did not have access to information that could identify individual participants during or after data collection. The study was performed in accordance with relevant guidelines and regulations. This was a retrospective review of patients’ files; therefore, a consent form was waived and not required."

**Data Collection**:

 - This sentence is confusing, and the use of parentheses is incorrect: "Baseline vital signs upon admission (heart rate, respiratory rate, oxygen saturation, systolic, and diastolic blood pressure) in addition to the most relevant laboratory parameters to anemia, such as (hemoglobin, hematocrit, ferritin, D-dimer, and alkaline phosphatase) were reviewed and analyzed."

 - *Recommendation*: Clarify and streamline the sentence: "Baseline vital signs upon admission, including heart rate, respiratory rate, oxygen saturation, and systolic and diastolic blood pressure, as well as key laboratory parameters related to anemia (hemoglobin, hematocrit, ferritin, D-dimer, and alkaline phosphatase), were reviewed and analyzed."

**Table 2**:

 - Issue: The row "Male 5.0% (120/2407) 1.58 1.00-2.51 0.028" **indicates a statistically significant adjusted odds ratio (aOR) with a confidence interval that includes 1.00**. This does not make sense to me, as a confidence interval that includes 1 should not be significant by definition. Please correct me if I am wrong or I am missing something about the analysis.

 - *Recommendation*: Verify the confidence interval and ensure that it does not include 1.00 if the result is statistically significant. If this is due to rounding, consider providing more precise values to clarify the significance.

**Table 3**:

 - The word "Normal" is followed by asterisks (*) that do not point to anything elsewhere in the table or text.

 - *Recommendation*: Remove the asterisks if they are not referencing any footnotes or additional information or provide a clear explanation if they are meant to reference something.

 - Inconsistent use of percentages in "Covid 19 outcomes Severity of anemia % (n/N) P-Value Moderate to Severe Disease Normal* 21.6% (590/2730) <0.001 Mild 30.3 (90/297) Moderate to Severe 29.3 (19/65)".

 - *Recommendation*: Ensure percentages are used consistently throughout the table for clarity. For example: "Mild 30.3% (90/297) Moderate to Severe 29.3% (19/65)".

**Results Section** (Referencing Table 3):

 - This sentence is problematic: "Upon classifying patients anemia into normal, mild and moderate to severe; severity of COVID-19 was higher in anemic compared to normal patients (p <0.001). On the other hand, moderate-to-severe anemia was associated with greater ICU admissions and mortality rates (p <0.001) as presented in Table 3."

 - **this is because a significant p-value indicates an overall difference in proportions among the groups as a whole (at least one of them is different, but we do not know which one) but does not specify which groups are different from each other**. Which specific group is different cannot be verified without further post-hoc testing which probably should come at the cost of a statistical penalty for repeated comparisons (Bonferroni, etc).

 - *Recommendation*: Clarify that the p-value here indicates an overall difference between subgroups and suggest that further post-hoc testing, such as Tukey's HSD, would be needed to identify specific group differences. Alternately perform such post-hoc testing and put the results in the table.

**Table 4**:

 - The confidence interval for "Gender (male) 1.43 1.15-178 <0.001 **" appears to be unusually wide**. I am wondering if this was due to a typographical error.

 - *Recommendation*: Verify the correct confidence interval and revise it if necessary. For example: "Gender (male) 1.43 1.15-1.78 <0.001".

**Discussion**:

 - Clarify this sentence:  "Based on the data collected from 3092 hospitalized adult patients in one of the largest hospitals in UAE, majority of the patients were males, 11.7% of whom were anemic."

 - *Recommendation*: Change "majority" to "the majority": "Based on the data collected from 3092 hospitalized adult patients in one of the largest hospitals in the UAE, the majority of the patients were males, 11.7% of whom were anemic."

 - Clarify this sentence: "Interestingly, while Chen et al. demonstrated an increasing anemia prevalence with age, the prevalence then decreased at age of 80 and above years old; however, a small number of cohort was reported in that study."

 - *Recommendation*: Change "a small number of cohort" to "a small cohort": "Interestingly, while Chen et al. demonstrated an increasing anemia prevalence with age, the prevalence then decreased at the age of 80 and above; however, a small cohort was reported in that study."

 - Clarify this sentence "The relationship between anemia and aging could be explained by common nutritional deficiencies (iron, folate, or vitamin B12), typically encountered with elderly or anemia of chronic diseases, and sometimes due to unknown reason."

 - *Recommendation*: Pluralize "reason" to "reasons": "The relationship between anemia and aging could be explained by common nutritional deficiencies (iron, folate, or vitamin B12), typically encountered in the elderly or in cases of anemia of chronic diseases, and sometimes due to unknown reasons."

 - The abrupt start of this sentence is confusing: "Whereas, females have relatively low ferritin levels, beginning to rise after menopause."

 - *Recommendation*: Simplify and clarify: "Females typically have relatively low ferritin levels, which begin to rise after menopause."

 - This sentence seems to be too strong. It is hard to say that a statistical model truly **proved** this: "This was further proven by the results of a multivariable logistic regression after adjustment for covariates."

 - *Recommendation*: Use a more appropriate term such as "supported" or "demonstrated": "This was further supported by the results of a multivariable logistic regression after adjustment for covariates."

 - Need a citation here: "Correspondingly, a single-center retrospective study assessed the impact of anemia on mortality rate among 333 hospitalized COVID-19 patients with anemia. The study has found that more than half of these patients were significantly at high risk of all-cause mortality."

 - *Recommendation*: Add a citation: "Correspondingly, a single-center retrospective study assessed the impact of anemia on mortality rate among 333 hospitalized COVID-19 patients with anemia. The study found that more than half of these patients were significantly at high risk of all-cause mortality (Citation needed)."

 - Wording is awkward here: "Considering other factors, multivariable regression analysis revealed that male gender, older age (≥48-year-old), significantly increased the severity of COVID-19 and ICU admissions. However, only older age was found to be an independent risk factor in addition to anemia for mortality."

 - *Recommendation*: Rework for clarity: "Multivariable regression analysis revealed that male gender and older age (≥48 years) significantly increased the severity of COVID-19 and ICU admissions. However, only older age, along with anemia, was found to be an independent risk factor for mortality."

 - I know what the authors mean here, but the way this sentence is worded is technically inaccurate. Our ability to interpret an association due to limitations in study design or analytic method do not actually affect the causal association itself, merely our ability to detect it: "This limited our interpretations and affected the causal association between anemia and COVID-19 outcomes."

 - *Recommendation*: Clarify that the limitations were a barrier to assessing causality: "This limited our interpretations and were a barrier to assessing the causal association between anemia and COVID-19 outcomes."

Is the work clearly and accurately presented and does it cite the current literature?

Yes

If applicable, is the statistical analysis and its interpretation appropriate?

Partly

Are all the source data underlying the results available to ensure full reproducibility?

Yes

Is the study design appropriate and is the work technically sound?

Yes

Are the conclusions drawn adequately supported by the results?

Partly

Are sufficient details of methods and analysis provided to allow replication by others?

Partly

Reviewer Expertise:

immunology, statistical methods

I confirm that I have read this submission and believe that I have an appropriate level of expertise to confirm that it is of an acceptable scientific standard, however I have significant reservations, as outlined above.

# Response

**Published:** 2024-10-29

**Authors:** salahdein aburuz

**Affiliations:** College of Medicine, United Arab Emirates University, Al Ain, Abu Dhabi, United Arab Emirates

**We truly appreciate the time and efforts you dedicated to reviewing our research work. Please note that all of your valuable comments have been addressed, and changes have been made in the manuscript accordingly. Many thanks**

**Response to comments:**

***Author List:***

***- The name "anan jarab" is not capitalized.***

***- Recommendation: Ensure consistent capitalization in the author list, e.g., "Anan Jarab”, unless there is a specific reason not to.***

*Response: Modified the name to: “Anan Jarab” as recommended.*

***Abstract:***

***Background:***

***- The sentence "This is the first study UAE aimed to assess the influence of anemia on COVID-19 severity, ICU admission, and mortality rate." is unclear.***

***- Recommendation: Revise to "This is the first study from the UAE aimed to assess the influence of anemia on COVID-19 severity, ICU admission, and mortality rate."***

*Response: Modified as recommended.*

***Methods:***

***- The term "retro-prospective chart review" is confusing.***

***- Recommendation: Use "retrospective chart review" for clarity.***

*Response: Modified as recommended.*

***Introduction:***

***- this is a run-on sentence and is hard to understand -  "According to the National Emergency Crisis and Disasters Management Authority (NCEMA), more than 700 thousand cases were confirmed, and 2135 deaths were reported in the United Arab Emirates (UAE) until November, 4th 2021 and the rate of developing new cases has increased to 67.0% by 2023 although majority of the people are vaccinated against COVID-19."***

***- Recommendation: Break this into two sentences for clarity: "According to the National Emergency Crisis and Disasters Management Authority (NCEMA), more than 700 thousand cases were confirmed, and 2135 deaths were reported in the United Arab Emirates (UAE) until November 4th, 2021. The rate of new cases increased by 67.0% by 2023, despite the majority of the population being vaccinated against COVID-19."***

*Response: Modified as recommended.*

***-This seems to be the wrong “basis”: "Genetic mutations of severe acute respiratory syndrome coronavirus-2 (SARS-COV-2) resulted in the emergence of more contagious variants; hence, increasing cases on daily bases."***

***- Recommendation: Correct the word "bases" to "basis": "Genetic mutations of severe acute respiratory syndrome coronavirus-2 (SARS-COV-2) resulted in the emergence of more contagious variants, hence increasing cases on a daily basis."***

*Response: Modified as recommended.*

***- There is redundant wording in this sentence: "Moreover, studies evaluating parameters of severe outcomes failed to demonstrate truly this association in a large considerable sample size."***

***- Recommendation: Simplify to avoid redundancy: "Moreover, studies evaluating parameters of severe outcomes failed to demonstrate this association in a large sample size."***

*Response: Modified as recommended.*

***Ethical Considerations:***

***- The wording here is problematic, as past and present tenses are mixed. The use of contractions is probably best avoided in a scientific paper: "Patients’ information was kept confidential and authors didn’t have access to information that could identify individual participants during or after data collection. Study was performed in accordance with relevant guidelines and regulations. This was a retrospective review patients’ files and therefore consent form is waived and not required."***

***- Recommendation: Revise for consistent tense and avoid contractions: "Patients’ information was kept confidential, and the authors did not have access to information that could identify individual participants during or after data collection. The study was performed in accordance with relevant guidelines and regulations. This was a retrospective review of patients’ files; therefore, a consent form was waived and not required."***

*Response: Modified as recommended.*

***Data Collection:***

***- This sentence is confusing, and the use of parentheses is incorrect: "Baseline vital signs upon admission (heart rate, respiratory rate, oxygen saturation, systolic, and diastolic blood pressure) in addition to the most relevant laboratory parameters to anemia, such as (hemoglobin, hematocrit, ferritin, D-dimer, and alkaline phosphatase) were reviewed and analyzed."***

***- Recommendation: Clarify and streamline the sentence: "Baseline vital signs upon admission, including heart rate, respiratory rate, oxygen saturation, and systolic and diastolic blood pressure, as well as key laboratory parameters related to anemia (hemoglobin, hematocrit, ferritin, D-dimer, and alkaline phosphatase), were reviewed and analyzed."***

*Response: Modified as recommended.*

***Table 2:***

***- Issue: The row "Male 5.0% (120/2407) 1.58 1.00-2.51 0.028" indicates a statistically significant adjusted odds ratio (aOR) with a confidence interval that includes 1.00. This does not make sense to me, as a confidence interval that includes 1 should not be significant by definition. Please correct me if I am wrong or I am missing something about the analysis.***

***- Recommendation: Verify the confidence interval and ensure that it does not include 1.00 if the result is statistically significant. If this is due to rounding, consider providing more precise values to clarify the significance.***

*Response: This was a system error, where all ORs were approximated to 2 decimal points. In this case, the value was 1.00095, that’s why, it was presented as 1.00.*

*So, we have modified it to be more precise.*

***Table 3:***

- The word "Normal" is followed by asterisks (*) that do not point to anything elsewhere in the table or text.

***- Recommendation: Remove the asterisks if they are not referencing any footnotes or additional information or provide a clear explanation if they are meant to reference something.***

Response: Removed the asterisks (*) as recommended.

- Inconsistent use of percentages in "Covid 19 outcomes Severity of anemia % (n/N) P-Value Moderate to Severe Disease Normal* 21.6% (590/2730) <0.001 Mild 30.3 (90/297) Moderate to Severe 29.3 (19/65)".

***- Recommendation: Ensure percentages are used consistently throughout the table for clarity. For example: "Mild 30.3% (90/297) Moderate to Severe 29.3% (19/65)".***

*Response: Percentages (%) has been modified to be consistent throughout the table as recommended.*

***Results Section (Referencing Table 3):***

***- This sentence is problematic: "Upon classifying patients anemia into normal, mild and moderate to severe; severity of COVID-19 was higher in anemic compared to normal patients (p <0.001). On the other hand, moderate-to-severe anemia was associated with greater ICU admissions and mortality rates (p <0.001) as presented in Table 3."***

***-this is because a significant p-value indicates an overall difference in proportions among the groups as a whole (at least one of them is different, but we do not know which one) but does not specify which groups are different from each other. Which specific group is different cannot be verified without further post-hoc testing which probably should come at the cost of a statistical penalty for repeated comparisons (Bonferroni, etc).***

***- Recommendation: Clarify that the p-value here indicates an overall difference between subgroups and suggest that further post-hoc testing, such as Tukey's HSD, would be needed to identify specific group differences. Alternately perform such post-hoc testing and put the results in the table.***

*Response: Modified as recommended.*

***Table 4:***

***- The confidence interval for "Gender (male) 1.43 1.15-178 <0.001" appears to be unusually wide. I am wondering if this was due to a typographical error.***

***- Recommendation: Verify the correct confidence interval and revise it if necessary. For example: "Gender (male) 1.43 1.15-1.78 <0.001".***

*Response: Corrected as recommended.*

***Discussion:***

***- Clarify this sentence:  "Based on the data collected from 3092 hospitalized adult patients in one of the largest hospitals in UAE, majority of the patients were males, 11.7% of whom were anemic."***

***- Recommendation: Change "majority" to "the majority": "Based on the data collected from 3092 hospitalized adult patients in one of the largest hospitals in the UAE, the majority of the patients were males, 11.7% of whom were anemic."***

*Response: Modified as recommended.*

***- Clarify this sentence: "Interestingly, while Chen et al. demonstrated an increasing anemia prevalence with age, the prevalence then decreased at age of 80 and above years old; however, a small number of cohort was reported in that study."***

***- Recommendation: Change "a small number of cohort" to "a small cohort": "Interestingly, while Chen et al. demonstrated an increasing anemia prevalence with age, the prevalence then decreased at the age of 80 and above; however, a small cohort was reported in that study."***

*Response: Modified as recommended.*

***- Clarify this sentence "The relationship between anemia and aging could be explained by common nutritional deficiencies (iron, folate, or vitamin B12), typically encountered with elderly or anemia of chronic diseases, and sometimes due to unknown reason."***

***- Recommendation: Pluralize "reason" to "reasons": "The relationship between anemia and aging could be explained by common nutritional deficiencies (iron, folate, or vitamin B12), typically encountered in the elderly or in cases of anemia of chronic diseases, and sometimes due to unknown reasons."***

*Response: Modified as recommended.*

***- The abrupt start of this sentence is confusing: "Whereas, females have relatively low ferritin levels, beginning to rise after menopause."***

***- Recommendation: Simplify and clarify: "Females typically have relatively low ferritin levels, which begin to rise after menopause."***

*Response: Modified as recommended.*

***- This sentence seems to be too strong. It is hard to say that a statistical model truly proved this: "This was further proven by the results of a multivariable logistic regression after adjustment for covariates."***

***- Recommendation: Use a more appropriate term such as "supported" or "demonstrated": "This was further supported by the results of a multivariable logistic regression after adjustment for covariates."***

*Response: Modified “proven” to “supported” as recommended.*

***- Need a citation here: "Correspondingly, a single-center retrospective study assessed the impact of anemia on mortality rate among 333 hospitalized COVID-19 patients with anemia. The study has found that more than half of these patients were significantly at high risk of all-cause mortality."***

***- Recommendation: Add a citation: "Correspondingly, a single-center retrospective study assessed the impact of anemia on mortality rate among 333 hospitalized COVID-19 patients with anemia. The study found that more than half of these patients were significantly at high risk of all-cause mortality (Citation needed)."***

*Response: Added citation number “8” for "Correspondingly, a single-center retrospective study assessed the impact of anemia on mortality rate among 333 hospitalized COVID-19 patients with anemia. The study found that more than half of these patients were significantly at high risk of all-cause mortality (Citation needed)."*

***- Wording is awkward here: "Considering other factors, multivariable regression analysis revealed that male gender, older age (≥48-year-old), significantly increased the severity of COVID-19 and ICU admissions. However, only older age was found to be an independent risk factor in addition to anemia for mortality."***

***- Recommendation: Rework for clarity: "Multivariable regression analysis revealed that male gender and older age (≥48 years) significantly increased the severity of COVID-19 and ICU admissions. However, only older age, along with anemia, was found to be an independent risk factor for mortality."***

*Response: Modified as recommended.*

***- I know what the authors mean here, but the way this sentence is worded is technically inaccurate. Our ability to interpret an association due to limitations in study design or analytic method do not actually affect the causal association itself, merely our ability to detect it: "This limited our interpretations and affected the causal association between anemia and COVID-19 outcomes."***

***- Recommendation: Clarify that the limitations were a barrier to assessing causality: "This limited our interpretations and was a barrier to assessing the causal association between anemia and COVID-19 outcomes."***

*Response: Modified as recommended.*

# Reviewer report: Reviewer response for version 1

**DOI:** 10.5256/f1000research.158636.r272010

**Published:** 2024-05-24

**Authors:** Joan-Lluis Vives-Corrons

**Affiliations:** 1 Red Cell Pathology and Haematopoietic Disorders (Rare Anaemias Unit), Institute for Leukaemia Research Josep Carreras (IJC), Barcelona, Spain

The manuscript provides valuable insights into the association between anemia and COVID-19 outcomes, supported by a large and well-characterized patient cohort. However, the limitations inherent in its retrospective, single-center design, and the lack of longitudinal and interventional data, suggest that further research is needed to confirm these findings and expand upon them. Future studies should aim for a prospective, multi-center approach, include longitudinal data, and consider the etiology of anemia to provide a more comprehensive understanding of this important health issue.

 Comments:

 1. The retrospective observational design limits the ability to control for all possible confounding variables and to establish a causal relationship. Prospective studies or randomized controlled trials would provide stronger evidence

 2. The study is conducted in a single center, which may limit the generalizability of the findings to other regions or healthcare settings. Multi-center studies would be more representative and enhance the external validity

 3.The study does not track changes in hematological parameters over time during hospitalization. Longitudinal data would provide insights into the dynamics of anemia and its potential evolution in response to COVID-19.

 4. The manuscript does not detail the clinical interventions and treatments related to anemia, which could influence patient outcomes. Understanding how anemia was managed during hospitalization would add depth to the analysis

 5. The study does not differentiate between types of anemia (e.g., iron deficiency vs. anemia of inflammation). This distinction could impact the interpretation of the findings, as different types of anemia might have different effects on COVID-19 outcomes

 6. While the study includes a diverse population in terms of gender and age, it does not address potential ethnic or socioeconomic factors that could influence both the prevalence of anemia and COVID-19 outcomes.

Is the work clearly and accurately presented and does it cite the current literature?

Yes

If applicable, is the statistical analysis and its interpretation appropriate?

I cannot comment. A qualified statistician is required.

Are all the source data underlying the results available to ensure full reproducibility?

Yes

Is the study design appropriate and is the work technically sound?

Yes

Are the conclusions drawn adequately supported by the results?

Yes

Are sufficient details of methods and analysis provided to allow replication by others?

Yes

Reviewer Expertise:

Rare Anemias

I confirm that I have read this submission and believe that I have an appropriate level of expertise to confirm that it is of an acceptable scientific standard, however I have significant reservations, as outlined above.

# Response

**Published:** 2024-10-29

**Authors:** salahdein aburuz

**Affiliations:** College of Medicine, United Arab Emirates University, Al Ain, Abu Dhabi, United Arab Emirates

**We truly appreciate the time and efforts you dedicated to reviewing our research work. Please note that all of your valuable comments have been addressed, and changes have been made in the manuscript accordingly. Many thanks**

***1. The retrospective observational design limits the ability to control for all possible confounding variables and to establish a causal relationship. Prospective studies or randomized controlled trials would provide stronger evidence***

*Noted. This has been discussed in the limitations of the study, as the authors were unable to conduct this research prospectively since the pandemic was an emergency crisis for all COVID-19 patients as well as healthcare providers.*****

***2. The study is conducted in a single center, which may limit the generalizability of the findings to other regions or healthcare settings. Multi-center studies would be more representative and enhance the external validity.***

*Noted. Since all COVID-19 patients that were included in this study were admitted to the ICU, it was a challenge that limited healthcare facilities treated such patients in the area, and all patients were sent to one center at the beginning of the crisis. We have also included this in the study limitation section.*****

***3.The study does not track changes in hematological parameters over time during hospitalization. Longitudinal data would provide insights into the dynamics of anemia and its potential evolution in response to COVID-19.***

*Noted. This has been discussed in the limitations of the study, as the authors were unable to conduct this research prospectively since the pandemic was an emergency crisis for all COVID-19 patients as well as healthcare providers. Additionally, Hospital stay wasn’t long enough to track changes. On the other hand, our goal was to see the impact of admission anemia on clinical outcomes.*****

***4. The manuscript does not detail the clinical interventions and treatments related to anemia, which could influence patient outcomes. Understanding how anemia was managed during hospitalization would add depth to the analysis.***

*During hospitalization, the treatment focused on the management of COVID-19, anemic patients were referred to specialists/consultants for further workup after their discharge. In case of severe anemia (HG <7), patients have received blood transfusions as per the guidelines, however, this information was not part of the data collection.*

***5. The study does not differentiate between types of anemia (e.g., iron deficiency vs. anemia of inflammation). This distinction could impact the interpretation of the findings, as different types of anemia might have different effects on COVID-19 outcomes***

*Noted. It was hard to differentiate the types of anemia as some patients may originally have had iron deficiency anemia and the condition worsened after the COVID-19 infection.*

***6. While the study includes a diverse population in terms of gender and age, it does not address potential ethnic or socioeconomic factors that could influence both the prevalence of anemia and COVID-19 outcomes.***

*This was the aim that has been discussed in another previously published study. It is also cited in this paper the discussion, Reference number 32.*

*AbuRuz S, Al-Azayzih A, ZainAlAbdin S, et al.: Clinical characteristics and risk factors for mortality among COVID-19 hospitalized patients in UAE: Does ethnic origin have an impact. PLoS One. 2022;17(3):e0264547. 35235580 10.1371/journal.pone.0264547 PMC8890645*
